# Lipid metabolism in the adrenal gland

**DOI:** 10.3389/fendo.2025.1577505

**Published:** 2025-06-09

**Authors:** Anika Aderhold, Vasileia Ismini Alexaki

**Affiliations:** Institute for Clinical Chemistry and Laboratory Medicine, Faculty of Medicine and University Hospital Carl Gustav Carus, Technische Universität Dresden, Dresden, Germany

**Keywords:** lipid metabolism, adrenal cortex, adrenal medulla, cholesterol metabolism, phospholipids, cortisol, aldosterone

## Abstract

The adrenal gland consists of the medulla and the cortex. The chromaffin cells of the adrenal medulla release catecholamines via regulated exocytosis. Vesicle formation, trafficking, maturation and fusion with the plasma membrane are orchestrated by lipids such as cholesterol, diacylglycerol, phosphatidic acid and phosphatidylinositol-4,5-bisphosphate. On the other hand, the adrenal cortex is a highly specialized lipid-metabolizing organ secreting steroid hormones. Cholesterol, acquired from circulating lipoproteins and *de novo* biosynthesis, is mobilized from intracellular stores and transported to mitochondria to be used as a substrate for steroidogenesis. Steroidogenesis is regulated by free polyunsaturated fatty acids (PUFA) and an increased PUFA content in phospholipids promotes steroidogenesis. Cholesterol efflux and lipid-processing macrophages further contribute to lipid homeostasis in the adrenal gland. Given that lipidomics have revolutionized our perception of cell function, we anticipate that this will also hold true for the investigation of adrenocortical function. Such investigations may pinpoint novel targets for the management of abnormal adrenal function.

## Introduction

The adrenal gland plays a pivotal role in vertebrate physiology and survival, as it mediates responses to danger and stress. It consists of the medulla, which releases catecholamines upon activation by splanchnic nerves in the so called ‘fight or flight response’, and the cortex, which secretes corticoid and other steroid hormones (1–3). Here, we summarize the role of lipids in the secretory function of chromaffin cells and we review the role of lipid metabolism in adrenocortical steroidogenesis.

## Lipids as regulators of catecholamine secretion

Catecholamines, i.e. adrenaline and nor-adrenaline, are released by chromaffin cells through a process that involves secretory vesicles budding off the Golgi apparatus, trafficking to the plasma membrane and their regulated exocytosis (4). Membrane lipid composition plays a key role in these processes (5). Lipid rafts in the Golgi membrane can guide protein clustering required for vesicle formation (5). Diacylglycerol (DAG), phosphatidic acid (PA), sphingolipids and cholesterol are implicated in fission of secretory vesicles (5). After formation, granules mature through acidification and condensation, and associate with actin to be transported to the plasma membrane, where catecholamines are secreted via regulated exocytosis (4, 5). Exocytosis requires vesicle docking, priming and Ca^2+^-dependent fusion with the plasma membrane. These processes involve the assembly of soluble N-ethylmaleimide-sensitive factor attachment protein receptor (SNARE) proteins, the synaptic vesicle VAMP (synaptobrevin), and the plasma membrane proteins syntaxin and synaptosomal-associated protein of 25Kda (SNAP-25) (4). Phospholipids, like lysophosphatidylcholine (LPC), and cone-shaped lipids, such as cholesterol, DAG and PA, play a critical role in this process by regulating protein assembly and driving negative membrane curvature, which facilitates the opening of the secretory pore (4–8). Also, increased phosphoinositide (PI) amounts in the plasma membrane and the secretory granules promote exocytosis. Particularly phosphatidylinositol-4,5-bisphosphate (PI(4,5)P_2_) localizes at sites of exocytosis, binds to proteins such as syntaxin-1, and promotes the actin-mediated conveyance of secretory granules to the plasma membrane (4, 9–12). Similarly, PA produced from phospholipids (such as phosphatidylcholine (PC), phosphatidylethanolmine (PE), phosphatidylglycerol (PG)) by phospholipase D1 (PLD1) or from DAG by diacylglycerol kinase, accumulates at the plasma membrane near exocytotic sites and contributes to lipid bilayer bending, binds to proteins like syntaxin-1, and promotes PtdIns(4,5)P_2_ production (4, 13). While monounsaturated PA increase the number of exocytotic events by eventually driving granule docking, polyunsaturated PA regulate fusion pore stability and expansion (14). DAG primes exocytosis via activation of protein kinase C and Munc13, which modulate the function of syntaxin isoforms (15). Polyunsaturated fatty acids (PUFAs) can also interact with syntaxin isoforms aiding SNARE complex formation (16, 17). Particularly arachidonic acid (AA) released from DAG and phospholipids during exocytosis increases SNARE complex formation and fosters granule docking and exocytosis (17, 18). During exocytosis, phospholipids are scrambled in the plasma membrane, with phosphatidylserine (PS) being externalized to the outer leaflet (19). PS clusters inhibit synaptotagmin 1 membrane penetration, which is required to promote fusion pore formation (20). Sphingosine, a releasable backbone of sphingolipids, activates vesicular synaptobrevin and promotes granule tethering (21, 22). Moreover, extracellular sphingosine-1-phosphate promotes exocytosis via activation of S1P receptors (23) and sphingomyelin derivatives enhance the frequency of fusion events in chromaffin cells (24). Finally, after completion of the secretory event, the granule membrane components are entirely recycled by DAG-driven endocytosis (25).

## Steroidogenesis in the adrenal cortex

The adrenal cortex consists of the *zona glomerulosa*, which produces aldosterone, and the *zona fasciculata* that produces glucocorticoids (1, 2). In primates, a third inner zone, the *zona reticularis*, produces the steroid hormone dehydroepiandrosterone (DHEA) and its sulfate ester (DHEA-S) (3, 26). Adrenocortical function is regulated by the hypothalamic–pituitary–adrenal (HPA) axis. Stress triggers the production of corticotropin-releasing-hormone (CRH) from the hypothalamus, which induces the release of adrenocorticotropic hormone (ACTH) from the anterior pituitary that reaches the adrenal gland via the circulation and binds to its receptor (melanocortin 2 receptor, MC2R) inducing corticoid production (27). ACTH is the exclusive stimulus for glucocorticoid release, while secretion of aldosterone is mainly induced by the renin-angiotensin-aldosterone system (RAAS) via angiotensin II and elevated circulating potassium levels (27, 28).

Corticoid hormones are not stored but synthesized *de novo* from cholesterol for immediate secretion. Glucocorticoid synthesis is triggered by binding of ACTH to MC2R, a G protein-coupled receptor (GPCR) activating the cyclic adenosine monophosphate (cAMP)-protein kinase A (PKA) signaling pathway. PKA activates hormone-sensitive lipase (HSL), which hydrolyzes cholesterol esters (CEs) stored in lipid droplets. Free cholesterol is transported through a complex mechanism involving steroidogenic acute regulatory (StAR) protein into mitochondria, where it serves as a substrate for steroid biosynthesis (2, 27). Cholesterol transport into mitochondria is the rate-limiting step of steroidogenesis (2, 27). Angiotensin II binds to the angiotensin type 1 receptor (AGT1R), triggering increase of intracellular calcium levels, which leads to activation of calmodulin kinase (CaMK). The latter induces StAR activation via its phosphorylation (29). Once inside the mitochondria, cholesterol is processed by cytochrome P450scc (P450 side-chain cleavage or CYP11A1), which cleaves cholesterol’s aliphatic side-chain, generating pregnenolone. CYP11A1 expression is induced by ACTH and angiotensin II via cAMP signaling (27, 30). Pregnenolone is transformed into progesterone by 3β-hydroxysteroid dehydrogenase (3β-HSD). Pregnenolone and progesterone generated in mitochondria transfer to the endoplasmatic reticulum (ER), where the next steps of steroidogenesis take place (31). In humans, progesterone is converted by CYP21 to 11-deoxycorticosterone, which is further metabolized to corticosterone by CYP11B1. Corticosterone is metabolized in the *zona glomerulosa* by CYP11B2 to aldosterone. In the *zona fasciculata*, progesterone is converted by CYP17 to 17-hydroxyprogesterone, which is processed by CYP21 to 11-deoxycortisol. In the final step of glucocorticoid synthesis, CYP11B1 converts 11-deoxycortisol to cortisol (27). While CYP11B1 is constitutively expressed in the *zona fasciculata*, CYP11B2 expression in the *zona glomerulosa* is regulated by circulating factors, such as angiotensin II, sodium and lipoproteins, like low-density lipoproteins (LDL), high-density lipoproteins (HDL) and very low-density lipoproteins (VLDL) (32–35). In the *zona reticularis*, 17-hydroxyprogesterone is converted by CYP17A1 to DHEA, which can be further metabolized to sex hormones (27).

## Cholesterol homeostasis in adrenocortical cells

**Figure 1:**
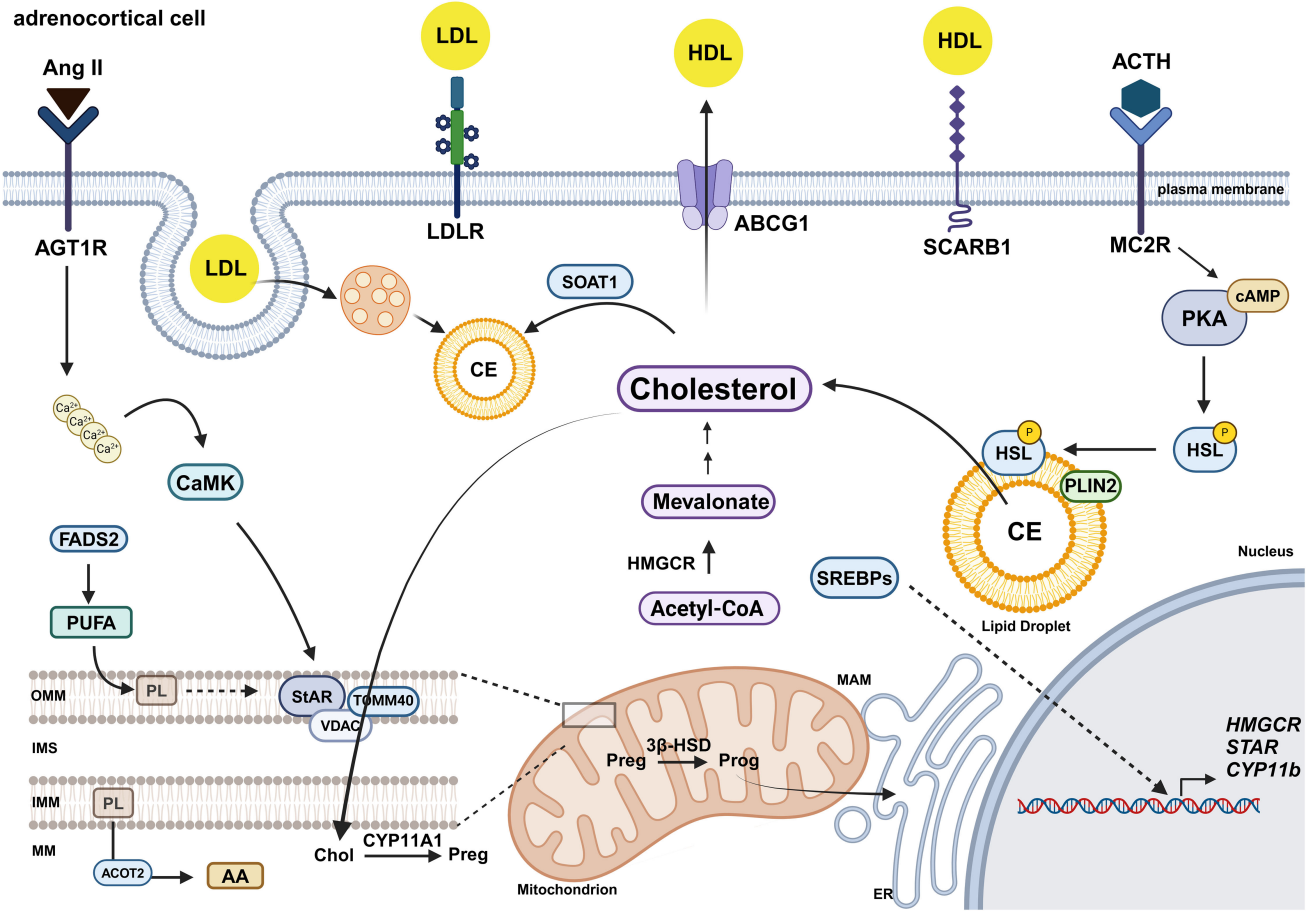
Lipid metabolism in adrenocortical cells. In adrenocortical cells, cholesterol derives from circulating lipoproteins and *de novo* biosynthesis. Low-density lipoproteins (LDLs) are internalized via the LDL receptor (LDLR) and endocytosis, while high-density lipoproteins (HDL) are taken up via scavenger receptor class B type I (SCARB1) (39, 40). Excess cholesterol is either esterified by SOAT1 and stored in lipid droplets (57) or exported via Adenosine triphosphate (ATP)-binding cassette transporter G1 (ABCG1) (85). Adrenocorticotropic hormone (ACTH) binding to melanocortin 2 receptor (MC2R) activates protein kinase A (PKA), which induces hormone-sensitive lipase (HSL) phosphorylation and translocation to the lipid droplets, assisted by perilipin 2 (PLIN2), promoting cholesterol release (66, 67). Angiotensin II (Ang II) binds to the angiotensin type 1 receptor (AGT1R), triggering the increase of intracellular calcium levels, leading to activation of calmodulin kinase (CaMK), which induces StAR activation (29). *De novo* synthesis of cholesterol is regulated by the rate-limiting conversion of acetyl-CoA to mevalonate via hydroxymethylglutaryl-CoA (HMG-CoA) reductase (HMGCR) (52). Gene expression of HMGCR and other cholesterogenic proteins is induced by Sterol regulatory element-binding proteins (SREBP) (53, 55). Free cholesterol is transported into mitochondria by steroidogenic acute regulatory (StAR) protein, through a complex process involving a number of different proteins, such as Voltage-dependent anion channels (VDAC) and translocase of the outer mitochondrial membrane 40 (TOMM40) (27, 31, 74–76, 107, 108). Cholesterol transfer from the ER to mitochondria is facilitated via ER-mitochondria contact sites, called mitochondria-associated membranes (MAMs) (31, 75). Fatty acid desaturase 2 (FADS2)-mediated increase in the PUFA content of mitochondrial phospholipids promotes cholesterol import into mitochondria (59). Moreover, arachidonic acid (AA) released from phospholipids by acyl-CoA thioesterase 2 (ACOT2) promotes steroidogenesis (96, 97). In mitochondria, steroidogenesis starts with the conversion of cholesterol to pregnenolone by CYP11A1 (27, 30).

Cholesterol serves as a precursor for steroid hormone production (36, 37). Cholesterol availability in adrenocortical cells is covered by 1. uptake from circulating lipoproteins 2. *de novo* synthesis and 3. CEs stored in lipid droplets (30, 37) (**Figure 1**). However, the exact contribution of these pathways to steroidogenesis and the flexibility in switching between them at baseline or stimulated conditions, are not fully understood. Excess intracellular cholesterol is transferred to circulating HDL through cholesterol efflux (38).

### Cholesterol acquisition from lipoproteins

Lipoproteins supply adrenocortical cells with cholesterol for steroid hormone production. LDL and HDL are internalized via binding to the LDL receptor (LDLR) and scavenger receptor class B type I (SCARB1), respectively, followed by endocytosis (37, 39, 40). Patients deficient for LDLR have normal serum cortisol levels but show reduced cortisol production in response to ACTH (41). Similarly, patients with SCARB1 mutation have normal cortisol concentrations, but reduced cortisol levels upon stimulation with an ACTH derivative (42). In contrast, LDLR and SCARB1 expression is increased in the adrenal cortex of patients with primary aldosteronism (43, 44). Accordingly, angiotensin II upregulates the expression of LDLR and SCARB1 (45). Besides providing cholesterol, lipoproteins (LDL, HDL, VLDL) trigger signaling events, including mobilization of intracellular calcium and cAMP response element binding (CREB) activation, thereby inducing expression of proteins involved in steroidogenesis, such as CYP11B2 and StAR (39, 44, 46, 47). LDLR is downregulated with aging in the adrenal cortex of primates limiting cholesterol uptake and DHEA-S secretion (48). Intriguingly, cholesterol uptake was shown to be dependent on autophagy, a mechanism mediating the degradation of cellular components (49). Autophagy disruption in Leydig cells leads to down-regulation of SCARB1, inefficient cholesterol supply and reduced testosterone production (49). Moreover, in Drosophila, autophagosomes sequester and transport cholesterol for steroid synthesis, while their disruption leads to cholesterol accumulation in lipid droplets (50).

### Cholesterol synthesis

Along with cholesterol imported from circulating lipoproteins, *de novo* biosynthesized cholesterol also fuels adrenocortical steroidogenesis. Acetyl-CoA, the precursor molecule of cholesterol, is produced in the cytosol by the ATP citrate lyase (ACLY) (51). The rate-limiting reaction of cholesterol synthesis is the conversion of acetyl-CoA to mevalonate by hydroxymethylglutaryl-CoA reductase (HMGCR) (52). Low sterol concentration is sensed by sterol regulatory element-binding proteins (SREBPs) that induce HMGCR expression (53). Through a cascade of reactions mevalonate is metabolized to squalene, which is processed in the ER membrane via lanosterol and desmosterol to cholesterol (52, 54). The central transcriptional activator of steroidogenesis, Steroidogenic Factor 1 (SF-1) binds to the promoter and induces the expression of several genes encoding for cholesterogenic proteins (55). Peripartum and lactation-associated adrenal gland plasticity in female rats involves downregulation of HMGCR expression and depletion of intra-adrenal cholesterol stores despite increased LDLR and SCARB1 expression; this is associated with basal hypercorticism and reduced responsiveness to ACTH, conferring postpartum anxiolysis (56). These evolutionary adaptations are overridden by feeding with a high-fat diet (HFD), which prevents the peripartum reduction of HMGCR expression and cholesterol stores (56).

### Cholesterol storage and mobilization

Uptaken or synthesized cholesterol is esterified by sterol O-acyltransferase 1 (SOAT1) with fatty acids and stored in lipid droplets, which makes cholesterol rapidly available for steroidogenesis (57). Inhibition of Acyl-coenzyme A: cholesterol acyltransferase (ACAT), which converts cholesterol to CE, reduces aldosterone production via suppression of CYP11B2 expression (58). Impaired steroidogenesis, as in congenital adrenal lipoid hyperplasia, or disruption of cholesterol mobilization and mitochondrial import, for instance due to HSL or StAR deficiency, lead to increased accumulation of CEs in lipid droplets (59–63). On the other hand, depleting the intracellular cholesterol pool in steroidogenic cells leads to lipid droplet shrinkage (64). Lipid droplets share contact sites with mitochondria and the ER, thereby facilitating immediate cholesterol transport to these organelles (31). Cholesterol mobilization occurs through lipophagy, where lipid droplets are engulfed by phagosomes followed by fusion with lysosomes, or through hormonally-controlled lipolysis mediated by HSL (63, 65). ACTH stimulation triggers via PKA HSL phosphorylation, which modestly increases HSL activity and, more importantly, directs HSL to lipid droplets, a process assisted by perilipin 2 (PLIN2), which resides on the lipid droplet surface (66, 67). PLIN2 deficiency in mice leads to pronounced lipid droplet accumulation in adrenocortical cells (67).

Impaired lipid mobilization and enhanced lipid accumulation in adrenocortical cells is accompanied by increased expression of macrophage markers, suggesting a role of adrenal gland macrophages in the local lipid turnover (67, 68). Similarly to lipid-associated macrophages (LAMs) present in other tissues, such as the adipose tissue, adrenal gland macrophages are rich in lipid droplets and present LAM signatures, including expression of Triggering receptor expressed on myeloid cells 2 (*Trem2*), Lipoprotein lipase (*Lpl*), *Cd9* and *Cd36* (68–70). Removal of adrenal gland macrophages causes increased lipid accumulation in the adrenal cortex (68). Consequently, adrenal gland macrophages regulate adrenocortical steroidogenesis in acute and chronic stress conditions, like cold exposure and atherosclerosis, respectively, through a mechanism dependent on TREM2 and macrophage-specific TREM2 deletion in mice increases serum glucocorticoid levels (70). These findings underscore the critical homeostatic role of macrophages in adrenocortical lipid metabolism and steroidogenesis.

### Cholesterol trafficking

Cholesterol levels are sensed by SREBPs residing in the ER (71). Cholesterol freed from CEs is transferred from lipid droplets to mitochondria by StAR. StAR mutations lead to impaired adrenal steroidogenesis, a condition termed congenital adrenal lipoid hyperplasia (72). StAR localizes at the outer mitochondrial membrane (OMM) and unfolds upon cholesterol binding at a C-terminal domain, a process requiring glucose regulatory protein-78 (GRP78) (73). Subsequently StAR mediates cholesterol transport into mitochondria through a complex and not entirely understood process involving a number of different proteins, such as Voltage-dependent anion channel 1 (VDAC1), VDAC2, mitochondrial transporter protein (TSPO), translocase of the outer mitochondrial membrane 40 (TOMM40) and GRP78 (27, 74–76). StAR-mediated cholesterol import depends on the efficiency of the electron transport chain and ATP production (77, 78). Moreover, cholesterol import into mitochondria is affected by the polyunsaturated fatty acid (PUFA) content of mitochondrial phospholipids. Reduced PUFA content in the phospholipids of mitochondrial membranes associates with diminished cholesterol import, mitochondrial membrane potential and oxidative phosphorylation (59). Accordingly, Acyl-CoA synthetase 4 (ACSL4), which inserts CoA to PUFAs facilitating their esterification into phospholipids, is highly expressed in the adrenal gland and required for steroidogenesis (59, 79). Cholesterol is thought to be transferred through direct contact sites connecting lipid droplets, mitochondria, the ER and the plasma membrane. Mitochondria, the ER, the plasma membrane, ER-mitochondria contact sites, so called mitochondria-associated membranes (MAMs) and plasma membrane-associated membranes (PAMs) have all unique and plastic lipid compositions (80). cAMP signaling triggers the formation of plasma membrane-ER and ER-mitochondria contacts (80). Cholesterol and proteins involved in cholesterol transfer accumulate at MAMs (31, 75). Aster proteins mediate cholesterol traffic from the plasma membrane to the ER and from the ER to mitochondria (81, 82). Furthermore, syntaxin (STX)-5 and a-SNAP mediate delivery of plasma membrane cholesterol to mitochondria (83, 84).

### Cholesterol efflux

Cholesterol levels in adrenocortical cells are regulated by cholesterol efflux mediated by ATP-binding cassette transporter G1 (ABCG1) and apolipoprotein E (ApoE) (85). Fasting stress reduces *Apoe* and *Abcg1* expression inhibiting cholesterol efflux (38). Adrenocortical ABCG1 deficiency leads to enhanced glucocorticoid production and, paradoxically, increased expression of genes encoding for proteins involved in cholesterol uptake, such as *Ldlr*, and cholesterol synthesis, such as *Hmgcr* and Squalene Epoxidase (*Sqle*) (85). ApoE-deficient mice present impaired cholesterol efflux associated with enhanced stress-induced glucocorticoid secretion (38). In contrast, cholesterol efflux is increased and adrenocortical steroidogenesis is reduced by synthetic HDL particles, which promote reverse cholesterol transport and thereby present a therapeutic strategy against atherosclerosis (86). Besides cholesterol homeostasis, lipoprotein release also serves long-range intercellular signaling mediated by proteins loaded onto lipoproteins. For instance, Sonic hedgehog (SHH), which is expressed in adrenocortical cells beneath the adrenal capsule, is released on lipoproteins, along with Hh pathway inhibitors regulating its long-range effects (87, 88).

## Sphingolipids and phospholipids as regulators of steroidogenesis

Apart from cholesterol metabolism and steroidogenesis, also other lipid metabolic pathways are dynamically regulated and play crucial roles in adrenocortical function. The adrenal gland presents organized spatial lipid distribution of sphingolipids and phospholipids (89). ACTH and cAMP signaling reduce the amounts of several sphingolipids, including sphingomyelin, ceramides, and sphingosine, induce sphingosine kinase activity and increase released S1P, while the latter promotes StAR, TSPO, LDLR and SCARB1 expression and glucocorticoid production (90, 91). Loss-of-function mutations in S1P lyase (SGPL1), lead to accumulation of sphingolipids in lysosomes and a condition termed sphingolipidose (92). Accordingly, *Sgpl1^-/-^* mice present disrupted adrenocortical zonation and impaired steroidogenic protein expression (92).

Moreover, phospholipids can determine the steroidogenic capacity of adrenocortical cells based on their PUFA content (59). AA is one of the most abundant acyl chains in PC, PE, PG and PI in the murine adrenal gland (59). Similarly, AA is among the most abundant lipids in the human adrenal cortex (59, 93). ACSL4-mediated esterification of free AA into phospholipids promotes steroidogenesis (79, 94, 95). On the other hand, hormonal stimulation and cAMP signaling induce acyl-CoA thioesterase 2 (ACOT2)-mediated release of AA from phospholipids into mitochondria (96, 97). Moreover, AA is metabolized by lipoxygenases to lipid mediators, such as hydroxyeicosatetraenoates, which can also regulate steroidogenesis (96).

The acyl chain composition of phospholipids in the adrenal gland is under dietary influence. For instance, AA-containing phospholipids increase in the adrenal gland of mice fed a HFD, aligning with enhanced corticoid output (59). Fatty acid desaturase 2 (FADS2), the rate-limiting enzyme of PUFA synthesis, is highly expressed in the adrenal gland and is upregulated in conditions of elevated corticoid synthesis, such as obesity or adrenal adenomas. Inhibition of FADS2 perturbs cholesterol transfer into mitochondria, mitochondrial function and steroidogenesis in adrenocortical cells, while steroidogenesis is partially restored by AA supplementation. In accordance, FADS2 deficiency in mice receiving a low-PUFA diet leads to reduced amounts of AA-containing phospholipids in the adrenal cortex, reduced glucocorticoid serum levels, enhanced lipid droplet accumulation and perturbed mitochondrial structure in adrenocortical cells. Accordingly, pharmacological inhibition of FADS2 reduces corticoid production in mice with established obesity (59). Moreover, the n-3 PUFA eicosapentaenoic acid (EPA) diminishes FADS2 expression and steroidogenesis in mouse and human adrenocortical cells and icosapent ethyl, an EPA analog, which is in clinical use for reduction of cardiovascular disease risk, efficiently reduces corticosterone and aldosterone serum levels in obese animals (59). Hence, treatment with dietary adjuncts, such as icosapent ethyl, could be an appealing strategy to clinically tackle dysregulated cortisol and aldosterone production.

## Diseases of disturbed lipid metabolism leading to impaired adrenocortical steroidogenesis

Disturbed lipid metabolism underlies several diseases manifested by adrenal insufficiency. Cortisol production is impaired in patients with LDLR deficiency or SCARB1 mutations (41, 42). Reduced HDL levels due to decreased hepatic lecithin-cholesterol acyltransferase (LCAT) activity in patients with liver cirrhosis associate with occurrence of relative adrenal insufficiency (RAI) (98). X-linked adrenoleukodystrophy (ALD), a disorder characterized by primary adrenal insufficiency, hypothyroidism and neurological symptoms, is caused by pathogenic variants of ABCD1, a very-long-chain fatty acid (VLCFA) transporter, leading to VLCFA accumulation in the form of CE (99–101). SGPL1 mutations are found in patients with steroid-resistant nephrotic syndrome (SRNS), which is characterized by adrenal insufficiency and chronic kidney disease (92, 102). Mutations in lysosomal acid lipase (cholesterol esterase) that hydrolyzes CE, lead to insufficient free cholesterol available to P450scc and development of Wolman disease (primary xanthomatosis) featured by adrenal insufficiency (103). Impaired cholesterol biosynthesis due to defects in sterol 7-reductase gene, DHCR7, in the Smith-Lemli-Opitz syndrome may lead to adrenal insufficiency, especially during times of stress or if LDL is inadequate (30).

## Discussion

The study of lipid metabolism has transformed our understanding of cell function. Lipids lie at the core of adrenal structure and function. Although the mechanisms involved in lipid metabolism in the adrenal gland were readily investigated during the past three decades, so far acquired knowledge has been barely therapeutically harnessed to clinically modulate adrenal function, for instance treat excessive cortisol or aldosterone production. However, lipophilic statin use in hypertensive and diabetic patients was associated with reduced basal and angiotensin II-stimulated aldosterone levels (104). Statins suppress HMGCR while they also inhibit caveolin-1-mediate endocytosis of lipoprotein receptors (104, 105). Hence, downregulation of aldosterone synthesis due to inhibition of cholesterol uptake and synthesis in adrenocortical cells may underlie the anti-hypertensive effect of statins (44). Especially lipophilic statins, such as simvastatin, which are more readily uptaken in the adrenal cortex, could be used to reduce aldosterone levels (104). Hence, preclinical and clinical studies and retrospective clinical analyses should be performed to elucidate the impact of statins in aldosterone release. As primary aldosteronism often co-occurs with cardiovascular disease and metabolic syndrome, combinatorial treatments with statins and antihypertensives could more potently reduce aldosterone levels and ameliorate the outcomes of primary aldosteronism, including ischemic heart disease or stroke (44, 106).

Moreover, modulation of the phospholipid composition of the adrenal cortex may present a means to control elevated corticoid production (59). Particularly, lowering the AA content of phospholipids through regulation of FADS2 by dietary adjuncts, such as icosapent ethyl, could be a novel strategy to regulate moderately elevated corticoid production in obesity, a concept which merits clinical investigation (59). Concluding, investigation of adrenal function under the prism of lipid metabolism may reveal new valuable concepts of endocrine regulation in normal and pathological conditions.
